# Optimization of a Microwave Assisted Extraction Method for Maximum Flavonols and Antioxidant Activity of Onion Extracts

**DOI:** 10.3390/antiox11122393

**Published:** 2022-12-02

**Authors:** Ana V. González-de-Peredo, Mercedes Vázquez-Espinosa, Estrella Espada-Bellido, Marta Ferreiro-González, Gerardo F. Barbero, Miguel Palma, Ceferino Carrera

**Affiliations:** Department of Analytical Chemistry, Faculty of Sciences, Agrifood Campus of International Excellence (ceiA3), University of Cadiz, IVAGRO, 11510 Puerto Real, Spain

**Keywords:** *Allium cepa* L., antioxidant activity, Box–Behnken design, flavonols, microwave-assisted extraction, onion, response surface methodology, UHPLC

## Abstract

Nowadays, consumers demand bioactive foods that have the potential to limit the risk of suffering from several medical conditions. Onions present these desirable capabilities owing to its high content in antioxidant bioactive compounds. This work has used a Box–Behnken design with a response surface methodology to determine the best conditions in which to extract the polyphenols that are found in onions. Two extraction methods—one for the extraction of total flavonols and another one intended to obtain extracts with the highest possible antioxidant activity—have been developed and optimized. The following factors have been studied: temperature, %methanol in water, solvent pH, and sample–solvent volumetric ratio. The optimal conditions for the extraction of flavonols were 93.8% methanol in water, pH 2, 50 °C extraction temperature and 0.2:17.9 g:mL sample–solvent ratio. The best antioxidant activity levels were registered when using 74.2% methanol in water, pH 2, 99.9 °C extraction temperature and 0.2:18.2 g:mL sample–solvent ratio. Both optimized methods used short extraction times, and presented good precision levels and successful results when used with an assortment of onion varieties. According to total flavonols and antioxidant activity data, with 7.557 ± 0.3261 and 12.08 ± 0.0379 mg g^−1^, respectively, the developed methods achieved comparable or even superior results to those obtained by other authors.

## 1. Introduction

Nowadays, both population and governmental entities increasingly demand the identification of those dietary patterns, specific bioactive foods and their components with the capacity to decrease the risk of suffering from several health disorders [1]. In this regard, secondary metabolites are substances produced by plants for their own growth and reproduction purposes, but they also present a substantial interest in human health [2]. Polyphenols are an important class within these natural metabolites because of their beneficial properties, and they can be found in many of the vegetables that are regularly consumed by humans [3]. Their most evident benefit to consumers is their capacity to inhibit oxidation [4]. Oxidation, or oxidative stress, is a disturbance characterized by an imbalance between the production and the accumulation of oxygen-reactive species (ROS) in cells and tissues against the ability of the biological system to counterbalance these reactive products [5]. This imbalance is associated with the induction of several medical chronic or degenerative diseases, such as cancer, as well as cardiovascular, neurological or respiratory disorders [5]. Polyphenols are potent antioxidants that can contribute to maintaining a homeostatic balance by protecting body cells against oxidative damage and preserving the body’s proper functioning [6]. For this reason, a polyphenol-rich diet is highly recommended, with an ideal daily intake of 1177 mg by men and 1192 mg by women [7]. Even though there is a high number of foods that have been praised for their health-promoting properties, those from the *Allium* genus have aroused a considerably special interest. *Allium* is a genus of monocotyledonous flowering plants that includes hundreds of species, with cultivated onions (*Allium cepa*), garlic (*Allium sativum*), shallots (*Allium ascalonicum*), leeks (*Allium porrum*) and chives (*Allium schoenoprasum*), among others.

This work focuses on the study of the onion (*Allium cepa* L.) and its polyphenol content, since this is a priority aspect in analytical and food chemistry [6]. Flavonoids, particularly quercetin glycosides, are the most remarkable polyphenols found in onions. Onions are one of the vegetables with the highest content of these compounds and the greatest flavonoid contributors to regular dietary patterns. The most abundant flavonoid forms in onions are quercetin 4-*O*-glucoside and quercetin 3,4-*O*-diglucoside, which account for over 85% of their total flavonoid content [8]. It is worth noting that the glycosylated forms of quercetin have superior properties to those exhibited by aglycones. Glycosidic derivatives are preferentially absorbed by humans when compared to quercetin aglycone [9]. These derivatives are the compounds that most confer *Allium cepa* L. with antimicrobial, antispasmodic, anticholesterolaemic, hypotensive, hypoglycaemic, antiasthmatic, anticancer and antioxidant properties [10].

Based on the already mentioned beneficial properties of the polyphenols found in certain foods, researchers have extensively studied specific techniques and conditions for the extraction of these compounds, whether for analytical or industrial purposes. Several techniques such as maceration, percolation, hydro-distillation, boiling, reflux, soaking or Soxhlet have been most frequently used. However, in addition to these techniques, other more advanced methods are increasingly being implemented to try and improve on the shortcomings of more conventional methods. Such limitations include the demand of large amounts of solvents, low extraction yields, poor selectivity, long extraction times, thermal degradation of the target compounds, as well as environment-related concerns or high costs [11]. According to an article previously published by our research group [12], using ultrasound-assisted methods for the extraction of flavonols from onion samples presents considerable advantages. Nevertheless, due to the diverse chemical composition of flavonols, no single standard method has been identified as ideal for the extraction of every specific flavonol. For this reason, in this work, Microwave-Assisted Extraction (MAE) is proposed as a suitable alternative technique for the extraction of flavonols from onions.

MAE is based on the changes that take place in the structure of onion cells when non-ionizing electromagnetic (EM) waves are applied at frequencies ranging from 300 MHz (radio-frequency range at lower frequency in the electromagnetic spectrum) up to 300 GHz (infrared at the higher frequency in the electromagnetic spectrum) [13]. This EM is converted into thermal energies through polar components interactions generating heat. So, MAE heats the entire solution (solvent and sample) to the required temperature in a short time. It also avoids the thermal gradient caused by other conventional heating methods, thus avoiding the risk of degrading the flavonols in the samples, since they are thermolabile bioactive compounds [14]. Flavonols are released from the onion matrix during microwave heating, as a considerable amount of pressure builds up, which enhances the matrix porosity and allows, in turn, better penetration of the extracting solvent. With regard to the Ultrasound-Assisted Extraction (UAE) method previously developed by our research team [12], other authors have also observed that MAE obtains similar or higher flavonol yields when applied to other natural matrices [15,16].

Furthermore, some articles have been found in the literature that use MAE [8,9,17,18,19,20,21], rather than more traditional and frequently used techniques such as sonication or centrifugation [22,23,24], for the extraction of flavonols from onions. Only one of these works investigates the influence that certain extraction variables, specifically extraction time and %ethanol in the extraction solvent, have on the extraction of flavonols [20]. The rest of these articles either do not carry out any optimization of the extraction method variables, or simply focus on the total phenolic compounds analyzed by spectrophotometric methods and not in flavonols. The study of extraction variables is of great importance, since having an efficient extraction technique such as MAE is not enough to obtain the best results. It is a fact that certain extraction variables, such as temperature, time or solvent have a substantial effect on the final extraction yields and must therefore be optimized for a fully satisfactory result. Response surface methodology (RSM), together with a design of experiments (DOE), are most frequently used for the optimization of extraction processes. RSM allows to model the statistical data and analyze how the response of interest (the dependent variables) is affected by specific factors (the independent variables).

Consequently, the present study intends to determine, on the one hand, the effect that four extraction factors (temperature, percentage of methanol in the solvent, solvent pH and sample–solvent volume ratio) have on the extracts’ flavonol content determined individually by Ultra-High-Efficiency Liquid Chromatography (UHPLC), and on the other hand, the antioxidant capacity of the extracts. For this purpose, a total of two experimental designs have been conducted, focusing on two of the most important characteristics of onions: their glycosylated quercetin content, and their antioxidant capacity. The results have been compared to those obtained by UAE to highlight the importance of having different extraction methods based on methanol/water mixtures as a solvent to evaluate the quality of the different onion varieties and to improve the selection of the final product thanks to its analytical quantification [25].

## 2. Materials and Methods

### 2.1. Onion Samples

An assortment of onion varieties has been employed in this study. Red onion, in particular, purchased from a local market in the province of Cadiz (Spain), was used for the optimization and development of the MAE methods. This variety was chosen because it is richer in flavonols than yellow or white onions [10]. Once the methods had been developed, a range of other varieties were also employed to verify the applicability of the method to other varieties of onions. Specifically, the following varieties were studied: spring white onion, French white onion, sweet white onion, white Chalota onion, yellow onion, purple onion and red Label onion. All the onion samples underwent a pretreatment that consisted of chopping the bulbs, freeze-drying in an LYOALFA freeze-dryer (Azbil Telstar Technologies, Terrassa, Barcelona, Spain) and grinding the resulting product using a GRINDOMIX blade mill (Retsch GM200, Haan, Germany). Finally, all the samples were stored in a freezer at −20 °C.

### 2.2. Chemical and Reagents

Two reagents were employed for the MAE procedure: HPLC-grade methanol (Fischer Scientifics, Loughborough, UK) and ultra-pure water obtained from a Milli-Q water-purification system (EMD Millipore Corporation, Bedford, MA, USA). Furthermore, a sodium hydroxide solution (NaOH, 1 M) and a hydrochloric acid solution (HCl, 1 M), both from Panreac (Barcelona, Spain) were used to adjust the pH of the extraction solvents. For the analysis and quantification of the flavonols in the extract, different reagents were employed. Milli-Q water, methanol, and formic acid (Merck KGaA, Darmstadt, Germany) were used for the identification of the flavonols by liquid chromatography coupled with mass spectrometry, while Milli-Q water, acetonitrile (Panreac, Barcelona, Spain) and acetic acid (Merck KGaA, Darmstadt, Germany) were employed for the quantification of the flavonols by ultra-high-performance liquid chromatography. Quercetin 3-*O*-glucoside supplied by Sigma-Aldrich (Steinheim, Germany) was the standard used for the quantification of the flavonols. The reagent DPPH (2,2-diphenyl-1-picrylhydrazyl) radical scavenging (Sigma—Aldrich, San Luis, MO, USA) was employed for the quantification of the antioxidant activity. The standard for the quantification of the antioxidant activity was 6-hydroxy-2,5,7,8-tetramethylchroman-2-carboxylic acid (Trolox) supplied by Sigma—Aldrich (Steinheim, Germany).

### 2.3. Microwave-Assisted Extraction

As mentioned above, a microwave-assisted extraction procedure was employed to obtain onion extracts with a large amount of total flavonols and high antioxidant activity. The MAE equipment specifically employed for this study was a MARS 6 One TouchTM Technology system (1800 W) (CEM Corporation, Matthews, NC, USA). The following protocol was followed for routine sample preparation: 0.2 g of the homogenized onion sample was weighed into 75 mL MARSXpress vessels (CEM Corporation), and then the extraction solvent was added to each vessel. The vessels were securely closed and placed inside the microwave oven with eight other tubes with the same methanol percentage and total volume of solvent. The variables to be considered for the study were adjusted to each experimental condition. The factors to be investigated in this study were within the following ranges: solvent (50:50–100:0% methanol:water), pH (2–7), temperature (50–100 °C) and sample:solvent ratio (0.2:10–0.2:20 g:mL). The initial extraction time remained constant at 5 min, plus an additional cooling period. The onion extracts were centrifuged at 1702× *g* for 5 min. The precipitate was centrifuged again after re-dissolving in 5 mL of the same extraction solvent. Both supernatants were added to a 25 mL volumetric flask, which was then filled up to the mark with the same solvent. Finally, the flask content was stored at −20 °C until further analysis.

### 2.4. Identification and Quantification of Flavonols

The method used to identify flavonoids and subsequently quantify their content in the onion samples had been previously developed by our research group [26]. Before both analyses, the extracts were filtered through a 0.20 µm nylon syringe filter (Membrane Solutions, Dallas, TX, USA), and 3 µL were injected into each extraction system.

For the identification of the flavonols in the onion samples, UHPLC coupled with a quadrupole-time-of-flight mass spectrometric system (Q-ToF-MS) (Xevo G2 QToF, Waters Corp., Milford, MA, USA) was employed. The general characteristics of the UHPLC-Q-ToF-MS method were the following: a C18 analytical column (1.7 µm, 2.1 × 100 mm., AQUITY UPLC CSH C18, Waters Corp., Milford, MA, USA), a binary solvent system (phase A, 2% solution of formic acid in water, and phase B, 2% solution of formic acid in methanol) and a flow rate of 0.4 mL min^−1^. The compounds identified according to their retention time and molecular weight were the following: quercetin 3,7,4′-*O*-triglucoside (2.873 min, *m*/*z* = 787.1421), quercetin 7,4′-*O*-diglucoside (4.610 min, *m*/*z* = 625.1396), quercetin 3,4′-*O*-diglucoside (5.151 min, *m*/*z* = 625.1398), isorhamnetin 3,4′-*O*-diglucoside (5.311 min, *m*/*z* = 639.1559), quercetin 3-*O*-glucoside (5.202 min, *m*/*z* = 463.0886), quercetin 4′-*O*-glucoside (5.543 min, *m*/*z* = 463.0873) and isorhamnetin 4-*O*’-glucoside (5.614 min, *m*/*z* = 477.1040).

**Figure 1:**
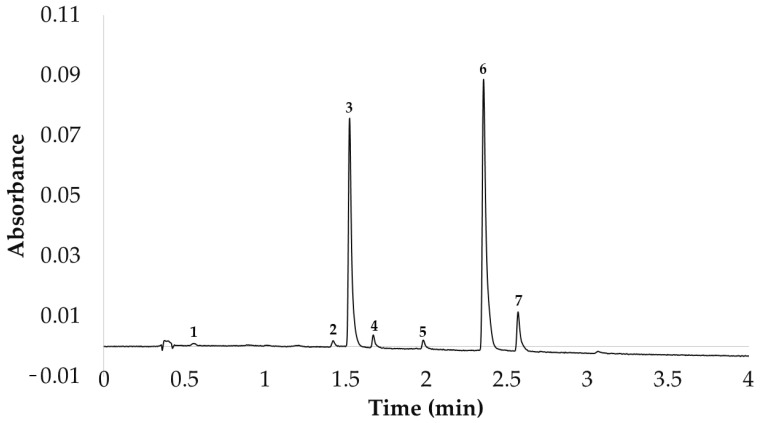
Representative red onion chromatogram at 360 nm. (**1**) Quercetin 3,7,4′-*O*-triglucoside; (**2**) quercetin 7,4′-*O*-diglucoside; (**3**) quercetin 3,4′-*O*-diglucoside; (**4**) isorhamnetin 3,4′-*O*-diglucoside; (**5**) quercetin 3-*O*-glucoside; (**6**) quercetin 4′-*O*-glucoside; (**7**) isorhamnetin 4′-*O*-glucoside.

After the flavonols in the red onion extracts had been identified, an UHPLC coupled with a photodiode array (PDA) detector (ACQUITY UPLC^®^ H-Class, Waters Corporation, Milford, MA, USA) was used for their separation and quantification. The general characteristics of the UHPLC-PDA method were the following: a C18 analytical column (1.7 µm, 2.1 × 50 mm., UPLC^®^ BEH C18, Waters Corporation, Milford, MA, USA), a binary solvent system (phase A, 2% solution of acetic acid in water, and phase B, 2% solution of acetic acid in acetonitrile), a flow rate of 0.6 mL min^−1^ and a column at 55 °C. The gradient employed, which had been previously optimized based on a multi-response (MRO) Box–Behnken (BBD) experimental design, allowed the separation and quantification of the seven flavonols that have been previously mentioned in less than 2.7 min [26]. The UHPLC chromatogram obtained can be seen in Figure 1. The flavonols were quantified by means of Empower3 Chromatography Data Software (Waters Corporation, Milford, MA, USA) with the PDA set at 360 nm. Specifically, a calibration curve of quercetin 3-*O*-glucoside (y = 13,884.19× − 19,765.16; R^2^ = 0.9991; 0.5–100 mg L^−1^) was constructed. The curve was built by injecting the quercetin 3-*O*-glucoside standard at different known concentrations (mg L^−1^) and measuring the area of the peak obtained (µV sec) (plot of peak areas versus concentrations). Since the other flavonols have similar chemical structures, and therefore similar absorbance, they were quantified based on this curve, but taking into account the molecular mass ratio of each compound. The sum of the extracted amounts of each of the flavonols identified has been expressed in this work as total flavonols (TF) and constitutes one of the response variables to be optimized.

### 2.5. Determining the Antioxidant Activity

The antioxidant capacity (electron donation) of the extracts obtained was measured as the loss of purple color of the DPPH radical solution. The molecules of α-diphenyl-*β*-picrylhydrazyl (DPPH; C_18_H_12_N_5_O_6_) change their optical absorption at 515 nm, and a pale-yellow color appears when the odd electron in its nitrogen atoms is reduced by the presence of antioxidant substances. For the routine determination of the antioxidant capacity of the samples, the following protocol was carried out: 100 μL of onion extract, previously filtered through a 0.45 µm nylon syringe filter (Membrane Solutions, Dallas, TX, USA), was mixed with 900 μL of 0.1 M Tris-HCl buffer (pH 7.4) and 2 mL of the DPPH solution (6 × 10^−5^ mol L^−1^ DPPH in methanol). The mixture was incubated (40 min) in the absence of light at room temperature and spectrophotometrically analyzed at 515 nm. This protocol was previously designed by Brand-Williams et al. [27]. The results were expressed as mg of Trolox equivalents (TE) per g of dry-weight sample (mg TE g^−1^ DW) using Trolox as standard solvent (y = 88.94 x + 0.7478; R^2^ = 0.9959; 0–1.4 10^−3^ mol L^−1^). Both supernatants were added to a 25 mL volumetric flask, which was then filled up using the same solvent.

### 2.6. MAE Optimization through Experimental Design

In this study, four factors or independent variables (X_1_: %methanol in water, X_2_: pH, X_3_: temperature and X_4_: ratio between sample and solvent) and two responses, or dependent variables (Y_TF_: extraction of total flavonols and Y_DPPH_: antioxidant activity of the extracts) have been investigated. Each of the response variables were first independently studied by applying BBD together with RSM. The purpose of using this spherical response surface methodology was to find out how MAE experimental parameters affect the extraction of flavonols, on the one hand, and the antioxidant activity of the extracts, on the other. Furthermore, one of the advantages of a Box–Behnken design is that the factors are in no case either all high or all low at the same time, i.e., no extreme combinations occur [28]. Subsequently, a MRO (Multi-Response Optimization) with the desirability function was simultaneously applied to both variables. MRO has the advantage that the two response variables can be adjusted to produce good yields from a single extraction. This is a very attractive feature from an economic point of view, since it represents a considerable saving with respect to the amount of solvent and length of time used.

For the individual optimization, three ranges were established for each factor, as follows: a lower level (−1), an intermediate level (0) and an upper level (1). The levels chosen for this study of onion matrices were selected considering the previous experiences of our research group, and can be seen in Table 1. On the other hand, because of the thermability of the bioactive compounds of interest, temperature was examined by means of an independent stability study, as described in Section 3.1. An analysis of variance (ANOVA) was applied to the results from this stability study and Tukey’s honest significant difference test (Tukey’s HSD) was used to determine the differences between the sample means at 95% confidence level.

According to the four factors and one response variable, a BBD design was produced consisting of 27 experimental points with 3 repetitions at the center point, which was randomly conducted. The whole experimental design matrix used can be seen in Table 2.

The RSM was applied to the results of the BBD to obtain a regression model and its corresponding second-order polynomial equation (Equation (1)), which allows the correlation of the independent variables with the response one. Furthermore, the suitability of the elaborated model was determined by ANOVA. All these statistical studies were performed by means of the software application Statgraphics Centurion version XVI (Warrenton, VA, USA).

(1) $$Y=\beta_{0}+\overset{k}{\underset{i=1}{\sum }}\beta_{i}X_{i}+\beta_{ii\;}X_{i}^{2}+\underset{i}{\sum }\overset{k}{\underset{i=1}{\sum }}\beta_{ij}X_{i}X_{j}+r$$

where *Y* represents the responses; *β*_0_ the model constant; *βi* the coefficient for each main effect; *β_ij_* the coefficient corresponding to the interactions between factor *i* and factor *j; β_ii_* the coefficient of the quadratic factors that represent the curvature of the surface; *X* each one of the factors studied; and *r* the residual value (random error).

In addition to conducting the individual optimization of each response, the desirability function (*D*) was used to perform the MRO. The objective of MRO is to maximize D, which is defined as the geometric average of each response variable (*Y_i_*) individual desirability function *(d_i_*). Thus, *d_i_* is obtained by converting the predicted values from each response surface into a scale-free value within the range 0–1. In order to compare the results obtained from each individual optimization and from the MRO, a two-tailed *t*-test was used while assuming normal distribution and the same variance. These procedures were also carried out by means of the software application Statgraphics Centurion version XVI (Warrenton, VA, USA).

## 3. Results and Discussion

### 3.1. Stability Study

**Figure 2:**
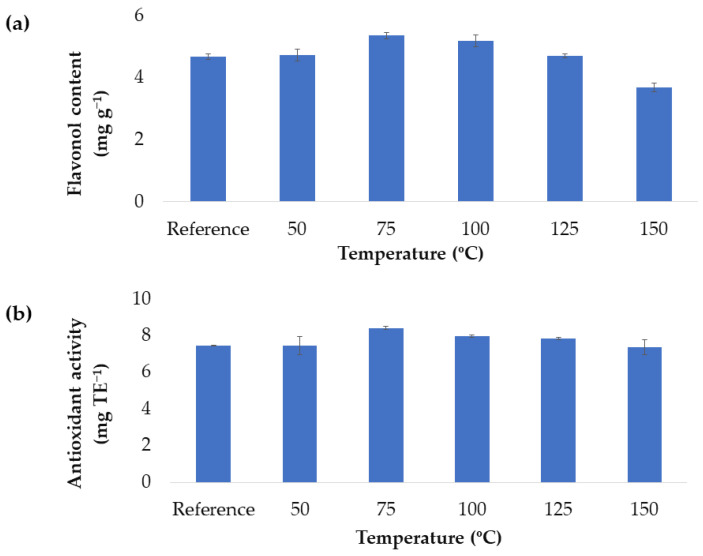
The effect from different temperature levels on the flavonols content (**a**) and on the antioxidant activity of the onion extracts (**b**). Each extraction was conducted in triplicate (mean ± SD) and the statically significant differences between the temperatures according to the Tukey’s test (*p*-value < 0.05) are indicated by the different letters over the corresponding column.

As mentioned above, the ranges of the independent variables were chosen based on previous experiences of our research group on the same natural matrix [12,28,29]. However, to determine the temperature ranges to be investigated, an additional study on the preservation of the antioxidant capacity of the extracts and its flavonol content when subjected to different temperatures was carried out. Although high temperatures generally promote large bioactive compound recoveries in solid–liquid extraction procedures, they may also promote the oxidative degradation of the compounds of interest [30]. Because of these contrary effects, the whole operational temperature range needs to be evaluated. For this purpose, first of all, a control extract was elaborated and subjected to intermediate extraction conditions (50:50 MeOH:H_2_O extraction solvent, 0.2:15 g:mL ratio and 20 min extraction time) but without any external temperature. Then, different samples from this control extract (15 mL to keep the same ratio) were subjected to different temperatures (10, 50, 75, 100, 125 and 150 °C) and the same intermediate conditions. The data corresponding to these extracts are shown in Figure 2a,b.

Based on the results obtained, it can be concluded that intermediate temperatures do not cause the degradation of the compounds of interest. However, when a certain temperature is reached, 125 °C for flavonols and 150 °C for antioxidant activity, there is a falling trend (according to Tukey’s test, they belong to different groups). This is probably due to the degradation of not only the flavonols, but also the rest of the compounds with antioxidant activity that can be found in the extracts. Based on these results and for the response variables to remain within the optimal region, it was decided to work during the Box–Behnken design in a temperature range between 50 and 100 °C.

### 3.2. Optimization of the MAE Method for Total Flavonols

With the BBD matrix finished, the RSM and ANOVA were applied to the data from the experimental extractions. Based on the ANOVA results (Table 3), it could be concluded that the analysis explains 96.77% of the total variability (R^2^ 96.77% and R^2^ adjusted 93.01%) and that the model fits well, as the lack of fit test presented a *p*-value (0.33) greater than 0.05.

To confirm the validity of the model, the ANOVA results included the coefficients for the parameters of the quadratic polynomial equation and their significance (*p*-values). Based on these significances, it is possible to know which parameters (factors and/or interactions between factors) have a relevant influence on the response (total flavonols extraction). The parameters with *p*-values lower than 0.05 were considered to have a relevant influence on the response at a 95% significance level.

In the case of the total flavonols, the linear variables percentage of methanol in the solvent (X_1_) and pH of the solvent (X_2_) showed *p*-values < 0.05, which indicates that both factors have a significant effect on the extraction of the flavonols. Furthermore, the interaction between both factors (MeOH-pH (X_1_X_2_)) also proved to have some influence on the response variable (*p*-value lower than 0.05). Regarding the quadratic interaction of the percentage of methanol (X_1_^2^), it also showed a relevant effect on the response (*p*-value < 0.0001). These results are consistent with those reported in the literature [10,11,12,13,14,15,16,17,18,19,20,21,22,23,24,25,26,27,28,29,30,31,32,33,34,35,36] and with those obtained in a previous study published by our research group on the application of UAE to onions [12], which indicate that the characteristics of the solvent used (pH and polarity) has a major influence on the extraction of these bioactive compounds from onion. Regarding the extraction of compounds such as the flavonoid glycosides that can be found in onion matrices, certain polar solvents, such as methanol or ethanol, have been used, since the compounds of interest are more soluble in this type of solvent [31]. However, since different flavonols may present varying polarity, no standardized ideal solvent composition can be applied to the extraction of every flavonol. Thus, the ideal methanol–water ratio to be used as the extraction solvent is generally, as already obtained in this study, one of the major factors that affect the extraction. In addition, polar solvents also contribute to improving heat transfer [36], as they can absorb microwave energy thanks to their high dielectric constant and dielectric loss [9]. In this regard, and for adequate microwave heating efficiency, solvent mixtures should be adjusted according to the dielectric constant that most favors the target extraction from each specific sample [37]. Finally, acidified solvents also promote bioactive compound extractions [38]. Acid solvents promote the breakdown of cell membranes, thus enhancing the release and solubilization of certain compounds [39].

**Figure 3:**
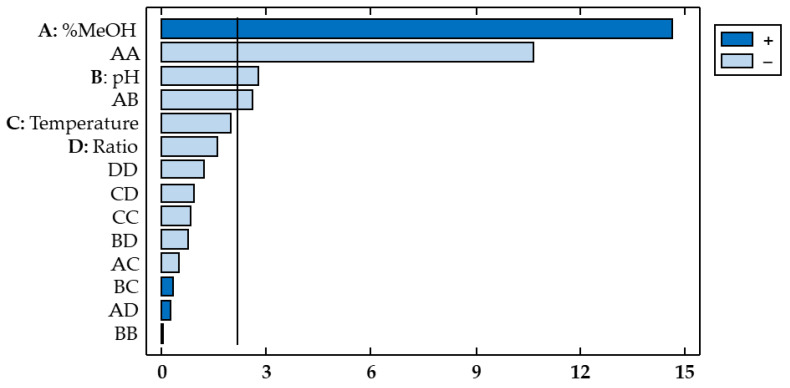
Pareto chart of the response variable total flavonols (Y_TF_).

A Pareto chart (Figure 3) was used for a more evident representation, where the horizontal bars corresponding to the relevant influencing factors or interactions cross the vertical reference line. In addition, the different colors indicate whether each factor has a positive or negative influence on the response variable. The percentage of methanol showed a positive effect on the response variable (b_1_ = 1.232), which indicates that by increasing the percentage of methanol in the solvent, the extraction of flavonols from red onion is enhanced. On the other hand, pH exhibited a negative effect (b_2_ = −0.2212), which indicates that flavonols are better extracted when acidified solvents are used.

Finally, based on the coefficients of the effects from the independent variables and their interactions (Table 3), a second-order mathematical equation can be built that predicts the total flavonols extracted (Y_TF_, response variable) as a function of the independent variables (Equation (2)):

***Y_TF_*** (mg g^−1^) = 6.468 + 1.232·X_1_ − 0.2212·X_2_ − 0.1450·X_3_ − 0.1176·X_4_ − 1.233·X_1_^2^ − 0.5124·X_1_X_2_ − 0.006252·X_1_X_3_ + 0.03175·X_1_X_4_ + 0.003131·X_2_^2^ + 0.04233·X_2_X_3_ − 0.09485·X_2_X_4_ − 0.08731·X_3_^2^ − 0.1162·X_3_X_4_ − 0.1276·X_4_^2^, (2)

**Figure 4:**
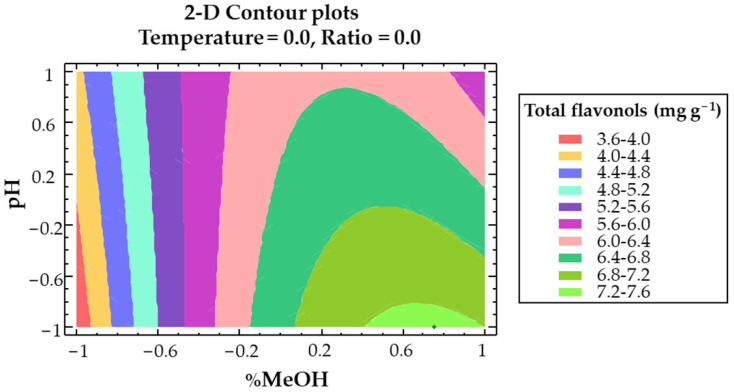
**A** 2D contour plot of the BBD using RSM to represent the effect of the solvent composition and pH on the Y_TF_ response variable.

A two-dimensional (2D) contour plot (Figure 4) using the fitted model was built to graphically represent the main effect from the interactions of the most influential parameters: effects from the methanol–pH interaction on the total flavonols recovery.

### 3.3. Optimization of the MAE Method for Antioxidant Activity

**Figure 5:**
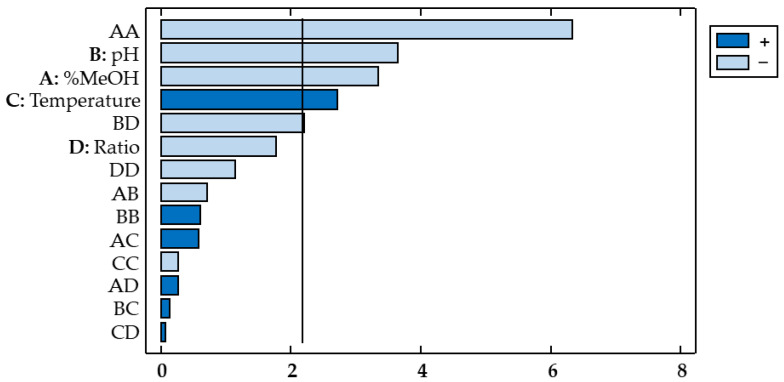
Pareto chart of the response variable antioxidant activity (Y_DPPH_).

In the case of the antioxidant activity, the RSM and ANOVA were also applied to the experimental matrix. The results obtained are shown in Table 4 and in the Pareto chart (Figure 5). According to these ANOVA results, the lack-of-fit test showed a *p*-value < 0.05, which indicates evidence of a lack of fit, i.e., the regression seems inadequate. This lack-of-fit value was expected, given the large number of substances of different natures that can influence the antioxidant activity of onions. This variation in the nature of the substances in onions, which is mainly attributable to their different polarities, generally results in lack of fit. In any case, the optimal conditions obtained are a compromise situation to obtain the most desirable antioxidant activity in the onion extracts [40]. Furthermore, it could be confirmed that the analysis explains 90.20% of the total variability (R^2^ 90.20% and R^2^ adjusted 78.77%).

With regard to the flavonols, the ANOVA and the Pareto chart allowed us to determine which factors and interactions between them had a more relevant (either positive or negative) influence on the antioxidant capacity of onions. In this case, the linear variables percentage of methanol in the solvent (X_1_), pH of the solvent (X_2_), and temperature (X_3_) showed *p*-values < 0.05, which indicate that these factors have a significant effect on the antioxidant activity of the onion extracts obtained. Regarding the influence from the interactions between factors, the interaction pH-ratio (X_2_X_4_) also showed a relevant influence on the response variable, with a *p*-value (0.047) lower than 0.05. Finally, the quadratic interaction percentage methanol–percentage methanol (X_1_^2^) also revealed a relevant effect on the response (*p*-value < 0.0001). As for the amount of total flavonols, solvent pH exhibited a negative effect on this response variable (b_2_ = −0.66), which results in greater antioxidant activity of the onion extract when lower solvent pH is used. As already mentioned, acid solvents facilitate the extraction of bioactive compounds through the rupture of cell membranes. On the other hand, the effect of the percentage of methanol on this response was contrary to that obtained for the flavonols. In the case of antioxidant capacity, decreases in the percentage of methanol favor the antioxidant capacity of the extracts (b_1_ = −0.64). This contrary trend is probably because flavonols are not the only compounds that determine the antioxidant capacity of the extracts. Thus, there are other bioactive compounds, such as anthocyanins, that also promote said antioxidant capacity and that can be better extracted using solvents of different characteristics. Finally, in this case, the temperature also affected the antioxidant capacity of the extracts. Specifically, temperature had a positive influence on this variable response (b_3_ = 0.45), which means that higher temperatures produced extracts with a greater antioxidant capacity. Like pH, higher temperatures favor the extraction of bioactive compounds, which results in extracts with greater antioxidant capacity. This is explained by the higher solubility of the target compounds in a warmer solvent, which achieves an improved mass transfer of the molecules of interest and a more efficient extraction [41].

**Figure 6:**
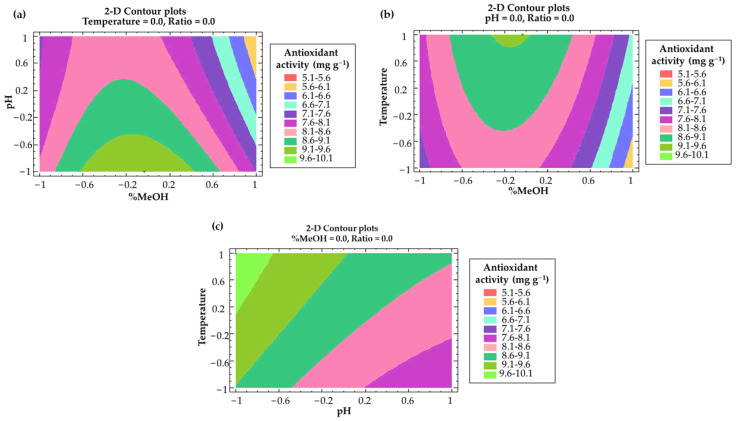
**A** 2D contour plot of the BBD using RSM to represent the combined effects of (**a**) solvent composition and pH; (**b**) solvent composition and temperature; (**c**) pH and temperature on the response variable antioxidant activity (Y_DPPH_).

Finally, the second-order mathematical equation (Equation (3)) intended to predict the antioxidant activity of the extracts (Y_DPPH_, dependent variable) as a function of the independent variables was built:

The fitted model was used to elaborate two-dimensional (2D) contour plots as a graphical representation of the main effects from the most influential parameters and their interactions. Figure 6 shows the effect from methanol–pH, methanol–temperature and pH–temperature interactions on the antioxidant activity of the extracts.

### 3.4. Optimal Conditions for the Individual Method

In addition to all the above-mentioned factors, the RSM should allow us to determine the values of the independent factors to obtain the best possible values of the response variables, i.e., the maximum extraction of flavonols, on the one hand, and the greatest antioxidant capacity of the extracts, on the other (Table 5).

Y_DPPH_ (mg g^−1^) = 8.7372 − 0.6405·X_1_ − 0.6663·X_2_ + 0.4522·X_3_ − 0.2948·X_4_ − 1.6590·X_1_^2^ − 0.3184·X_1_X_2_ + 0.1641·X_1_X_3_ + 0.07450·X_1_X_4_ + 0.1587·X_2_^2^ + 0.03630·X_2_X_3_ − 0.6355·X_2_X_4_ − 0.06580·X_3_^2^ + 0.02100·X_3_X_4_ − 0.2766·X_4_^2^, (3)

Given that, as acid solvents achieve greater yields and with higher antioxidant activity, pH 2 was established as the optimum value. However, regarding the percentage of methanol, the response variables presented opposite trends. Thus, although the optimal percentage of methanol for the extraction of flavonols was as high as 93.8%, with respect to the antioxidant activity of the extracts, the best results were obtained using 74.2% of methanol. This evidences that there are other compounds, apart from flavonols, that also provide antioxidant activity to onion extracts. Temperature was another independent variable that presented opposite trends so that the greatest flavonol yields were obtained at the lowest value of the range studied (50 °C), while the highest antioxidant activity was registered at the upper end of the temperature range (99.9 °C).

### 3.5. Extraction Time, Repeatability and Applicability to Different Onion Varieties

**Figure 7:**
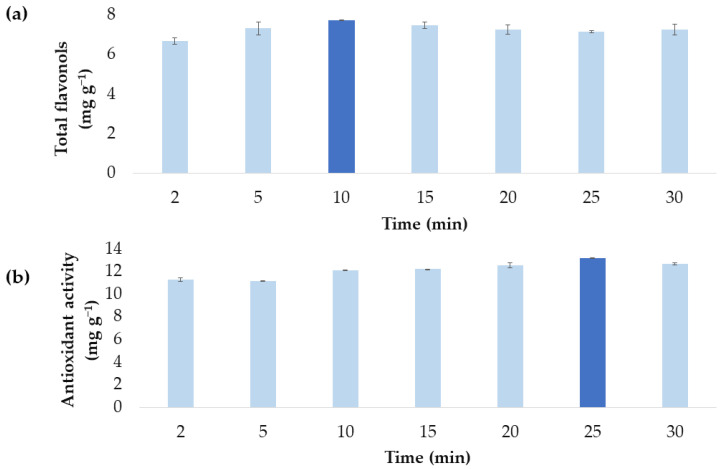
Effect of the extraction times on each optimized MAE method: (**a**) optimized MAE method for the maximum extraction of total flavonols and (**b**) optimized MAE method for extracts with maximum antioxidant activity. Each extraction time was used in three tests (mean ± SD) and the statically significant differences between the times according to Tukey’s test are indicated by the different letters over the corresponding column.

After each optimal factor had been established, the influence from different extraction times on each optimized MAE method was determined. For this purpose, each optimized extraction method was tested in triplicate for 2, 5, 10, 15, 20, 25 and 30 min extraction times. The test data can be seen in Figure 7.

From Figure 7a, it can be concluded that 5 min allows the largest yields of total flavonols (7.557 ± 0.3261 mg g^−1^). A shorter time (2 min) produces smaller yields, since the extraction process probably cannot be completed in such a short time. Longer extraction times do not show statically significant differences according to Tukey´s test. So, for rather similar yields, the best choice would be the one which demands the least time and energy.

It can also be concluded from Figure 7b that 10 min produces the onion extracts with the highest antioxidant activity (12.08 ± 0.03788 mg TE g^−1^); while, as in the case of total flavonols, no significant differences can be noticed as the time taken grows longer, according to Tukey´s test.

Once the optimum extraction times had been established, the precision of each method was studied in terms of repeatability and intermediate precision. Both aspects were evaluated by analyzing the coefficients of variance (CV): 30 experiments were conducted on three consecutive days to determine their intermediate precision, and 10 experiments were conducted on a single day to determine their repeatability. If both CVs are less than 5%, the method can be considered precise according to the AOAC for this type of assessment [42]. The MAE method for total flavonols showed the following CVs for repeatability and intermediate precision, respectively: 2.051% and 3.012%. The MAE method for antioxidant activity showed the following CVs for repeatability and intermediate precision, respectively: 3.980% and 4.561%. So, both methods can be considered precise.

Finally, both optimized and validated MAE methods were applied to several onion varieties to verify their efficacy in onions with different chemical compositions. Thus, 15 types of onion of different color, origin and variety were studied. The results regarding individual flavonols, as well as TFs, correspond to the optimized MAE method for the extraction of TF, and the results regarding antioxidant activity correspond to the optimized MAE method for maximum antioxidant activity. The results obtained are shown in Table 6.

It can be concluded that both MAE methods, for the extraction of total flavonols and to obtain onion extracts with high antioxidant activity, could be successfully applied to many onion varieties, which confirms their applicability to the production of extracts from onions with different chemical compositions. Such differences in chemical composition can be observed, for example, in the purple varieties, which contain, apart from flavonols, anthocyanins (a type of compounds that are only found in red/purple vegetables) in their matrices, and their extracts exhibit greater antioxidant activity.

### 3.6. Comparison against Other Extraction Methods

As previously mentioned, other authors have reported on different extraction methods to obtain flavonol-rich extracts, as well as extracts with high antioxidant activity. Our research group carried out a study on the development of a UAE method for these goals. As a summary of the results obtained in this study, our data have been compared against those reported by other studies, so that the efficiency of MAE can be appreciated.

It should first be pointed out that many of these studies employed traditional techniques for onion extraction purposes. Ren F. et al. [17] carried out a homogenization and shaking of the onion bulbs overnight (Red Baron and Hyskin varieties). By employing these extraction techniques, they reported 5.61 ± 0.27 mg g^−1^ and 0.90 ± 1.42 mg g^−1^ of total flavonols (sum of the individual flavonols analyzed by HPLC) in each variety, respectively. Regarding antioxidant activity, they reported 0.44 ± 0.01 mg g^−1^ and 0.01 ± 0.00 mg g^−1^ in each variety employing DPPH Antioxidant Power Assay. Majid I. et Nanda V. [18] centrifuged different varieties of onion bulbs for 15 min. They identified five individual flavonols by HPLC and reported the following total flavonols in an assortment of onion varieties: 212.37 ± 2.76, 199.10 ± 2.14, 279.50 ± 5.3, 302.80 ± 2.66, 359.80 ± 5.52, 440.20 ± 7.94, 149.50 ± 3.50 and 147.60 ± 2.45 mg kg^−1^. Traditional extraction techniques required long extraction times (in many cases, up to 24 h) but did not achieve large recoveries.

Apart from the traditional techniques, some articles found in the bibliography also investigated microwave-assisted extraction applied to onion matrices. In this regard, even though Zill-e-Huma et al. [8,21], Kumar B. et al. [9] and Soltoft M. et al. [22] employed MAE, they did not carry out any optimization of the extraction factors by DOE with RSM. They simply optimized some of the factors individually. According to the HPLC analyses carried out by Zill-e-Huma et al., flavonols were obtained at amounts between 3.30 mg g^−1^ and 0.04 mg g^−1^, depending on the onion variety studied. Kumar B. et al., reported 2.09 mg g^−1^ obtained from the onions’ edible outer layers and apical trimmings of red onion, and Soltoft M. et al., reported 0.46 mg g^−1^ from onion bulbs. Even though using an efficient extraction technique such as MAE is very important, correct optimization of the extraction factor is also vital. When the most suitable conditions for each specific purpose are applied, it is possible to maximize the recovery of the compounds of interest, minimize the extracted adjunct compounds and limit the degradation or alteration of the natural state of the extracts.

It should also be mentioned that three articles have been found in the bibliography that use MAE in combination with an experimental design. Thus, Jin E.Y. et al. [20] carried out a Central Composite Design (CCD) with RSM. These authors specifically studied how the percentage of ethanol and time affect the extraction of quercetin, as determined by HPLC. These studies reported extractions of 4.84 ± 0.14 mg g^−1^ quercetin from onion skin. Dairi S. et al. [23] and Pal, C.B. et Jadeja, G.C. [24], similarly to the method employed in this work, used a Box–Behnken design. However, these authors used the total phenolic compounds as the response variable determined by the Folin–Ciocalteu method. So, the MAE method developed in this work achieved yields comparable to or even greater than those obtained by the above-mentioned authors. This is probably attributable to the greater number of factors that have been considered for the DOE and to the chromatographic analysis used.

Finally, it is rather interesting to compare the results obtained in this study against our previous work, where UAE was used as the extraction method [12]. The red onion samples employed were alike, and the extractions and analyses were carried out at the same moment, employing the two optimized developed methods: UAE and MAE, respectively. On the one hand, the UAE method that had been developed for total flavonols could extract 8.193 ± 0.2500 mg g^−1^ flavonols from red onion bulbs, employing a solvent at 79% methanol content, pH 2, 60 °C temperature and 10 min extraction time. On the other hand, the onion extracts obtained through the UAE method could only reach antioxidant activity of 11.45 ± 0.05592 mg g^−1^ of TE from the red onion bulbs by employing a solvent at 77% methanol, pH 2, 59 °C temperature and 10 min of extraction time. First, it should be highlighted that the optimized MAE method used greater percentages of methanol stretching close to 100% for flavonols’ maximum yields. This fact had already been observed when UAE and MAE were compared against each other about total phenolic compounds (TPC) or total anthocyanins (TA) yields [28,29]. This coincidence led to the conclusion that, regardless of the bioactive compound of interest, MAE applied to onions is more efficient when using high methanol percentages. This is probably explained by, as previously mentioned, the dielectric constant of methanol, which enhances the solvent-heating efficiency of the microwaves. It should also be borne in mind that MAE is more efficient when used to extract compounds with a greater affinity for methanol than to water (according to the rule of equal polarity between solvent and compounds to be extracted that has been previously explained). Secondly, and based on the specific results obtained, it can be concluded that UAE achieves greater flavonol yields than MAE. This is mainly due to the two major compounds in onions: flavonol 3. quercetin 3,4′-*O*-diglucoside and flavonol 6. quercetin 4′-*O*-glucoside, which are more easily extracted by UAE (3.517 ± 0.04120 and 3.661 ± 0.04810 mg g^−1^, respectively) than by MAE (2.932 ± 0.03603 and 3.053 ± 0.02494 mg g^−1^, respectively), probably due to the greater amount of polar solvent used for UAE. However, regarding antioxidant activity, this is higher in the extracts obtained by MAE. This is explained by the larger amounts of CFT and TA extracted by MAE when compared against those obtained by UAE. Since bioactive compounds with electron donation capacity are more efficiently extracted by MAE rather than by UAE, MAE extracts present a greater content of these compounds and, logically, superior antioxidant activity.

### 3.7. Multi-Response Optimization

In addition to the optimization of each individual method, a multi-response optimization was also conducted. Multi-response optimization allows the simultaneous optimization of the two methods according to all the factors considered. MRO provides optimal results for both response variables with a single extraction, which represents a considerable saving in costs and time. The optimum conditions established were the following: 84.9% methanol in water as the extraction solvent, with a pH of 2.5, 50 °C extraction temperature, and 0.2:10 g:mL sample–solvent ratio. By applying these extraction conditions to the same red-onion sample for 10 min, onion extracts with the following characteristics were obtained: 7.63 ± 0.00 mg g^−1^ total flavonols and 12.05 ± 0.24 mg Trolox equivalent g^−1^. Assuming that the values follow a normal distribution and that there is no difference between the variances, a two-tailed *t*-test was applied to compare these results with those obtained by applying the individual methods using the same extraction time. Regarding TFs, it can be stated that there is a statistically significant difference between the means of the two variables (7.72 ± 0.02 mg g^−1^ of the individual optimization vs. 7.63 ± 0.00 mg g^−1^ of the multi-response optimization) because the *p*-value of the *F*-test is less than 0.05. Regarding the antioxidant activity, the result obtained by applying the multi-response optimization (12.08 ± 0.24 mg Trolox equivalent g^−1^) does not differ from the one obtained when using the individually optimized method (12.05 ± 0.24 mg Trolox equivalent g^−1^) and according to the *F*-test (the *p*-value was 0.89). These results confirm that the multi-response optimization method would be suitable for their application in quality control analytical laboratories, where time and costs must be minimized [43].

## 4. Conclusions

This research has successfully developed rapid and efficient techniques to obtain onion extracts rich in bioactive compounds. An extensive study of the relevant literature revealed that the MAE methods that have been developed in this work achieved yields comparable to or even greater than those obtained by the other authors. Specifically, the Box–Behnken design together with Response Surface Methodology has been applied to the optimization of three microwave-assisted extraction methods. The first MAE method was optimized for the extraction of the seven majority flavonols (Y_TF_) that can be found in onion bulbs; the second MAE method was optimized to produce onion extracts with high antioxidant activity (Y_DPPH_), and, finally, one multi-response optimized MAE method should allow good results to be obtained for both response variables (Y_TF_ and Y_DPPH_) in a single extraction. The multiple-response method presents some very interesting advantages based on solvent, cost and time savings. All the methods developed showed better results with a high percentage of methanol and an acid pH as extraction solvent. Furthermore, the MAE methods required short extraction times for good recoveries and showed good precision (CVs < 5%) and suitable applicability to different onion varieties.

## Figures and Tables

**Table 1:** Ranges studied for each MAE factor.

Factors	Studied Ranges
Methanol in water (%)	50–75–100
pH	2–4.5–7
Temperature (°C)	50–75–100
Ratio (g:mL)	0.2:10–0.2:15–0.2:20

**Table 2:** BBD design matrix, as well as experimental and predicted values for both response variables (Y_TF_ and Y_DPPH_).

Run	Factors				Responses			
	X_1_	X_2_	X_3_	X_4_	Y_TF_ (mg g^−1^)		Y_DPPH_ (mg g^−1^)	
					Experimental	Predicted	Experimental	Predicted
1	0	−1	0	0	6.506	6.692	9.102	9.559
2	1	−1	0	0	7.310	7.204	7.624	7.578
3	0	1	0	0	6.382	6.245	8.427	8.233
4	1	1	0	0	5.842	5.737	5.661	5.615
5	0	0	−1	−1	6.438	6.400	7.948	8.259
6	0	0	1	−1	6.406	6.342	8.997	9.121
7	0	0	−1	1	6.425	6.397	7.731	7.627
8	0	0	1	1	5.928	5.874	8.864	8.573
9	0	0	0	0	6.579	6.468	8.803	8.737
10	−1	0	0	−1	4.239	4.025	8.152	7.812
11	1	0	0	−1	6.205	6.426	5.661	6.382
12	−1	0	0	1	4.098	3.726	7.304	7.073
13	1	0	0	1	6.191	6.254	5.111	5.941
14	0	−1	−1	0	7.076	6.792	8.985	9.077
15	0	1	−1	0	6.302	6.265	7.706	7.679
16	0	−1	1	0	6.545	6.418	9.458	9.909
17	0	1	1	0	5.940	6.060	8.324	8.655
18	0	0	0	0	6.276	6.468	8.796	8.737
19	0	−1	0	−1	6.496	6.588	9.572	8.942
20	0	1	0	−1	6.331	6.335	9.071	8.887
21	0	−1	0	1	6.303	6.542	9.948	9.623
22	0	1	0	1	5.759	5.910	6.905	7.026
23	−1	0	−1	0	3.640	3.998	6.993	7.365
24	1	0	−1	0	6.558	6.587	6.399	5.756
25	−1	0	1	0	3.606	3.833	7.742	7.941
26	1	0	1	0	6.274	6.172	7.804	6.988
27	0	0	0	0	6.575	6.468	8.744	8.737

**Table 3:** ANOVA results for the Y_TF_ response variable.

Source	Source Code	Coefficients	Sum of Squares	Degrees of Freedom	Mean Square	*F*-Value	*p*-Value
A−Methanol	X_1_	1.232	13.8	1	13.8	214.2	<0.0001
B−pH	X_2_	−0.2212	0.4894	1	0.4894	7.6	0.0174
C−Temperature	X_3_	−0.1450	0.2523	1	0.2523	3.92	0.0712
D−Ratio	X_4_	−0.1176	0.1661	1	0.1661	2.58	0.1343
AB	X_1 × 2_	−0.5121	0.4375	1	0.4375	6.79	0.023
AC	X_1_X_3_	−0.06252	0.01564	1	0.01564	0.24	0.6311
AD	X_1_X_4_	0.03175	0.004032	1	0.004032	0.06	0.8067
BC	X_2_X_3_	0.04233	0.007166	1	0.007166	0.11	0.7445
BD	X_2_X_4_	−0.09485	0.03599	1	0.03599	0.56	0.4692
CD	X_3_X_4_	−0.1162	0.05399	1	0.05399	0.84	0.378
A^2^	X_1_^2^	−1.233	7.337	1	7.337	113.9	<0.0001
B^2^	X_2_^2^	0.003131	4.63 × 10^5^	1	4.63 × 10^5^	<0.0001	0.9791
C^2^	X_3_^2^	−0.08731	0.04357	1	0.04357	0.68	0.4269
D^2^	X_4_^2^	−0.1273	0.0926	1	0.0926	1.44	0.2537
Residual			0.773	12	0.06442		
Lack of Fit			0.7125	10	0.07125	2.36	0.3347
Error			0.06051	2	0.03025		
Total			23.96	26			

**Table 4:** ANOVA of the response variable antioxidant activity (Y_DPPH_).

Source	Source code	Coefficients	Sum of Squares	Degrees of Freedom	Mean Square	*F*-Value	*p*-Value
A−Methanol	X_1_	−0.6405	3.729	1	3.728	11.26	0.0057
B−pH	X_2_	−0.6632	4.398	1	4.398	13.28	0.0034
C−Temperature	X_3_	0.4522	2.454	1	2.454	7.41	0.0185
D−Ratio	X_4_	−0.2948	1.043	1	1.043	3.15	0.1013
AB	X_1_X_2_	−0.3184	0.1689	1	0.1689	0.51	0.4887
AC	X_1_X_3_	0.164	0.1076	1	0.1076	0.33	0.5791
AD	X_1_X_4_	0.0745	0.0222	1	0.0222	0.07	0.8001
BC	X_2_X_3_	0.03625	0.005256	1	0.005256	0.02	0.9018
BD	X_2_X_4_	−0.6355	1.615	1	1.615	4.88	0.0474
CD	X_3_X_4_	0.021	0.001764	1	0.001764	0.01	0.943
A^2^	X_1_^2^	−1.659	13.29	1	13.29	40.12	<0.0001
B^2^	X_2_^2^	0.1587	0.1188	1	0.1188	0.36	0.5603
C^2^	X_3_^2^	−0.06579	0.02474	1	0.02474	0.07	0.7893
D^2^	X_4_^2^	−0.2766	0.4372	1	0.4372	1.32	0.273
Residual			3.974	12	0.3312		
Lack of Fit			3.972	10	0.3972	382.3	0.0026
Error			0.002078	2	0.001039		
Total			40.56	26			

**Table 5:** Optimal conditions for individual methods.

Factor	Total Flavonols (Y_TF_)	Antioxidant Activity (Y_DPPH_)
Methanol (%)	93.8	74.2
pH	2	2
Temperature (°C)	50	99.9
Ratio (g mL^−1^)	0.2:17.9	0.2:18.2

**Table 6:** Application of both optimized MAE methods to different onion varieties.

Onion Variety	Peak 1 (mg g^−1^)	Peak 2 (mg g^−1^)	Peak 3 (mg g^−1^)	Peak 4 (mg g^−1^)	
Spring white I	0.3021 ^a,b,^* ± 2.764 10^−3^	0.2514 ^a^ ± 0.004759	2.205 ^a,b^ ± 0.04053	0.2942 ^a,b,c,d^ ± 0.002775	
French white	0.3112 ^a,b^ ± 3.731 10^−3^	0.2836 ^a^ ± 0.001730	4.151 ^c^ ± 0.01331	0.3338 ^d,e,f^ ± 0.001608	
Sweet white I	0.2835 ^a,b^ ± 7.731 10^−3^	0.3290 ^a^ ± 0.02380	0.3046 ^d^ ± 0.009464	0.2647 ^a^ ± 0.009279	
Spring white II	0.2927 ^a,b^ ± 0.004240	0.2411 ^a^ ± 0.002681	1.6706 ^a^ ± 0.03110	0.2744 ^a,b^ ± 0.00004783	
Sweet white II	0.2877 ^a,b^ ± 0.0008064	0.2457 ^a^ ± 0.0005264	1.206 ^a,d^ ± 0.01950	0.2771 ^a,b^ ± 0.0007797	
White Chalota	0.3156 ^b^ ± 0.01481	0.3273 ^a^ ± 0.01787	4.490 ^c^ ± 0.1826	0.3652 ^f^ ± 0.008562	
Sweet white III	0.2823 ^a,b^ ± 0.008634	0.2389 ^a^ ± 0.009181	1.674 ^a^ ± 0.02221	0.2875 ^a,b,c^ ± 0.005632	
Yellow I	0.2943 ^a,b^ ± 0.002428	0.2532 ^a^ ± 0.001529	2.324 ^a,b^ ± 0.008642	0.2810 ^a,b^ ± 0.003865	
Yellow II	0.2863 ^a,b^ ± 0.002395	0.2495 ^a^ ± 0.0009032	1.661 ^a^ ± 0.01582	0.2927 ^a,b,c,d^ ± 0.001744	
Yellow III	0.2862 ^a,b^ ± 0.001097	0.2411 ^a^ ± 0.0009408	1.324 ^a,d^ ± 0.002966	0.2863 ^a,b,c^ ± 0.0001668	
Purple	0.3045 ^a,b^ ± 0.009872	0.2704 ^a^ ± 0.02074	3.373 ^b,c,e^ ± 0.1420	0.3112 ^b,c,d,e^ ± 0.03485	
Red Label	0.3668 ^c^ ± 0.01955	3.1448 ^b^ ± 0.1222	0.3855 ^d^ ± 0.009240	0.3240 ^c,d,e,f^ ± 0.01219	
Red I	0.3053 ^a,b^ ± 0.0008264	0.2795 ^a^ ± 0.007189	3.597 ^c,e^ ± 0.0153	0.3425 ^e,f^ ± 0.003215	
Red II	0.3002 ^a,b^ ± 0.01513	0.2979 ^a^ ± 0.01639	2.932 ^b,e^ ± 0.008206	0.3644 ^f^ ± 0.004789	
Red III	0.3036 ^a,b^ ± 0.01132	1.482 ^c^ ± 0.006595	0.3291 ^d^ ± 0.008545	0.3132 ^b,c,d,e^ ± 0.009470	
**Onion Variety**	**Peak 5** **(mg g** **^−1^)**	**Peak 6** **(mg g** **^−1^)**	**Peak 7** **(mg g** **^−1^)**	**TF** **(mg g** **^−1^)**	**DPPH** **(mg g** **^−1^)**
Spring white I	0.2158 ^a^ ± 0.001627	1.6756 ^a^ ± 0.017358	0.3096 ^a^ ± 0.002412	5.254 ^a,b^ ± 0.02835	11.43 ^a,b^ ± 0.4508
French white	0.1950 ^a^ ± 0.002625	2.571 ^b,c^ ± 0.02157	0.3681 ^b,c^ ± 0.003582	8.213 ^c,d^ ± 0.005018	11.00 ^a,b^ ± 0.5310
Sweet white I	0.6154 ^b^ ± 0.01086	0.2537 ^d^ ± 0.006445	0.4532 ^d^ ± 0.001795	2.504 ^e^ ± 0.006578	8.594 ^a^ ± 0.2042
Spring white II	0.2008 ^a^ ± 0.000300	1.264 ^e^ ± 0.0004395	0.2522 ^e^ ± 0.0004585	4.196 ^a,f,g^ ± 0.03677	8.037 ^a^ ± 2.615
Sweet white II	0.1918 ^a^ ± 0.002727	1.096 ^e^ ± 0.009229	0.3018 ^a,e^ ± 0.0006869	3.606 ^e,f^ ± 0.01318	9.176 ^a,b^ ± 0.5467
White Chalota	0.2278 ^a^ ± 0.004061	2.761 ^c,f^ ± 0.03442	0.4413 ^d^ ± 0.007938	8.928 ^d^ ± 0.2702	11.48 ^a,b^ ± 0.6065
Sweet white III	0.1905 ^a^ ± 0.006503	1.372 ^e^ ± 0.02328	0.3240 ^a,b,c^ ± 0.007456	4.370 ^a,f,g^ ± 0.08289	12.01 ^a,b^ ± 6.8096
Yellow I	0.2255 ^a^ ± 0.0001763	2.642 ^c^ ± 0.0001432	0.2527 ^e^ ± 0.0002509	6.272 ^b^ ± 0.0002503	13.33 ^a,b^ ± 1.158
Yellow II	0.2008 ^a^ ± 0.00008577	1.108 ^e^ ± 0.0003443	0.3004 ^a,e^ ± 0.001571	4.099 ^f,g^ ± 0.009635	15.14 ^a,b^ ± 2.163
Yellow III	0.1883 ^a^ ± 0.001001	1.119 ^e^ ± 0.008392	0.3177 ^a,b^ ± 0.001829	3.762 ^f^ ± 0.006656	15.16 ^a,b^ ± 0.2260
Purple	0.2281 ^a^ ± 0.04692	2.778 ^c,f^ ± 0.2365	0.3368 ^a,b,c^ ± 0.04692	7.602 ^c^ ± 0.696	14.69 ^a,b^ ± 0.05138
Red Label	0.2034 ^a^ ± 0.01023	2.750 ^c^ ± 0.1259	0.3720 ^c,f^ ± 0.01094	7.547 ^c^ ± 0.3103	14.8 5 ^a,b^ ± 0.03486
Red I	0.2098 ^a^ ± 0.002913	2.293 ^b,g^ ± 0.01043	0.4210 ^d,f^ ± 0.008497	7.448 ^c^ ± 0.1879	12.67 ^a,b^ ± 0.01302
Red II	0.2358 ^a^ ± 0.007627	3.053 ^f^ ± 0.01911	0.5381 ^g^ ± 0.006105	7.722 ^c^ ± 0.02930	13.17 ^a,b^ ± 2.315
Red III	0.2104 ^a^ ± 0.006773	2.1497 ^g^ ± 0.06002	0.2929 ^a,e^ ± 0.006072	5.081 ^a,g^ ± 0.04278	17.87 ^b^ ± 0.4158

^a^ Peak 1. Quercetin 3,7,4′-*O*-triglucoside; Peak 2. quercetin 7,4′-*O*-diglucoside; Peak 3. quercetin 3,4′-*O*-diglucoside; Peak 4. isorhamnetin 3,4′-*O*-diglucoside; Peak 5. quercetin 3-*O*-glucoside; Peak 6. quercetin 4′-*O*-glucoside; Peak 7. isorhamnetin 4′-*O*-glucoside. * Each extraction was performed in triplicate (mean ± SD). Different letters within the same column mean that there is a statistically significant difference according to Tukey´s test at a significance level of 95%.

## Data Availability

The data presented in this study are contained within the article.
